# Analysis of Falls from Height Variables in Occupational Accidents

**DOI:** 10.3390/ijerph182413417

**Published:** 2021-12-20

**Authors:** María del Carmen Rey-Merchán, Jesús M. Gómez-de-Gabriel, Antonio López-Arquillos, Sang D. Choi

**Affiliations:** 1 PhD Program Advanced Computing, Energy and Plasmas, University of Cordoba, 14071 Cordoba, Spain; ep2remem@uco.es; 2 System Engineering and Automation Department, University of Malaga, 29071 Malaga, Spain; jesus.gomez@uma.es; 3 Economics and Business Management Department, University of Malaga, 29071 Malaga, Spain; 4 Department of Occupational and Environmental Safety and Health, University of Wisconsin-Whitewater, Wisconsin, WI 53190, USA; chois@uww.edu

**Keywords:** fall from height, accident, safety, worker, fatal

## Abstract

Fall-from-height accidents are linked to severe and fatal consequences for impacted workers. A better understanding of the related variables is necessary to improve worker safety. This study analyzed all fall-from-height occupational accidents recorded in Spain from 2009 to 2019, selected significant variables, and evaluated the influence concerning the seriousness of the falls from height. Based on a total of 290,583 fall-from-height accidents, the study shows that a male inexperienced worker in a small company working in a non-habitual workplace is more likely to suffer fatal consequences once the accident happens. An improved knowledge of fall-from-height accidents will improve safety conditions. The workers should be trained and informed about their specific risk depending on the variables analyzed. Safety training should consider more risky profiles. Results from the current study can help identify suitable fall prevention and risk mitigation actions in safety programs for companies.

## 1. Introduction

Fall-from-height (FFH) accidents are a cause for concern in occupational settings because they can affect worker health and productivity [1]. In this sense, the figures for fatal work injuries show a general upward trend [2]. The occurrence of FFH accidents was found to be higher among specific occupations, such as construction workers, Truck drivers, wholesale retail workers, and agriculture workers [3]. Various authors from different countries, such as the USA [4], China [5], Spain [6], or Korea [7], have studied FFH accidents to arrive at a better understanding of occupational accidents [8]. Currently, many safety strategies can mitigate the risk of FFH accidents, such as prevention through design, collective protection (safety barriers, scaffolding, nets, and guardrails), and personal protective equipment (e.g., safety harnesses) [9,10]. The appropriate use of FFH preventive measures is regulated by rules, such as by OHSA, European Directives, or similar national regulations. Unfortunately, despite the legal requirements, often workers do not use safety measures properly because of discomfort, restriction of movements or low risk perception, and FFH accidents continue to happen [10].

Several causation factors for FFH accidents have been identified in the literature. such as human behavior, safety management, activity, and worksite conditions [11,12]. Human behavior plays a significant role in FFH accidents. Overconfidence, misjudgment of the height, or carelessness are the major causes of FFH deaths [13]. Variables, such as gender [14] or age [15] of the worker, have been identified as influencing variables in occupational accidents. In recent research, it was found that women involved in an occupational accident had a longer duration of lost work time than men [16]. Regarding with age of the worker, in a review of previous literature [17], it was found that young workers had a higher injury rate for non-fatal accidents than older workers. In contrast, an analysis of fatal occupational injuries indicated that young workers had a lower fatality rate than older workers. In specific research about fatal FFH accidents in the construction industry, it was pointed out that workers older than 55 were represented disproportionately in the sample of incidents. These results suggested that declining physical and sensory capabilities are a factor in FFH accidents [12].

Organizational variables are one of the influential variables in FFH accidents. For instance, in a case-control study about factors related to fatal occupational injuries, temporary workers showed an increased risk of fatality after an occupational injury [18]. Other authors highlighted that the risks for temporary workers are much higher with some hazards but it depends on the task. For instance, the risk of a fall from a mobile scaffold is about 16 times higher, but for a fall on the same level, the risk rate is only 1.3 times higher [19]. In similar research developed in Singapore about FFH accidents during roof construction, self-employed workers had a higher rate of injury than non-self-employed workers [20]. The disparity might be caused by a number of factors, like self-employed workers tend to work alone and longer and they are not required to use fall protection devices.

Company size showed a relationship with the accident rates linked to FFH [21]. Small companies showed lower levels of training courses, proper safety measures, and personal fall arrest systems in comparison with big companies [22,23,24]. In research carried out in the USA about FFH accidents for roofers, it was found that roofers who worked at small companies employing less than 11 employees were more likely to have falls [25].

Work-site conditions are another relevant factor that was previously studied. In general, occupational accidents that take place out of the usual occupational setting, or workers conducting non-habitual tasks, showed an increased risk of a fatal result [18]. On one hand, surface conditions are especially important for workers performing tasks at elevated heights. An unexpected slippery surface or lack of illumination can be enough to cause n FFH accident [26,27]. In certain activities, workplace conditions can be affected by the weather and environmental conditions. In this sense, agriculture, farm, and construction workers face hot, humid, rainy, or windy weather conditions [28,29]. Illumination and weather conditions are determined by the hour of the day. It is remarkable that previous authors pointed that nighttime construction is more hazardous than daytime construction [30].

A similar group of factors have been studied previously by other authors for a better understanding of occupational accidents. On one hand, Camino et al. used cited variables collected from accidents officially recorded in Spain to study construction accidents and occupational accidents with ladders [6,31]. On the other hand, previous authors based their accident research on surveys in different countries [32,33]. However, a lack of updated research focused on the study of occupational FFH accidents that considers workers from different sectors was detected. Therefore, the aim of current research is to obtain new, extended and updated insights into the likely causes of FFH accidents in Spain to improve worker safety.

## 2. Materials and Methods

### 2.1. Data Accidents

According to Spanish Law, since 2003, every occupational accident should be recorded in the electronic system called Delt@ (Declaración Electrónica de Trabajadores Accidentados). Then every accident would be officially recorded and included in the cited database. For the current research, the Ministry of Labor and social security supplied all the accidents recorded from 2009 to 2019. Accidents recorded from 2003 to 2008 were not considered because their NACE codes (Nomenclature statistique des activités économiques dans la Communauté européenne) are different in cited period of time. A total number of 290,583 accidents due to falling from heights from all sectors was supplied. However, it is possible that some FFH remain unreported and avoided mandatory reporting. The distribution of the accidents is partially shown in Table 1. The categories not included in the table had very low values. It can be observed that 50 percent of the recorded FFH accidents were concentrated in only 15 categories from the 269 included in the NACE classification. Although construction codes were on the top of the ranking, other activities, such as moving or cleaning services, presented concerning data.

**Figure 1:**
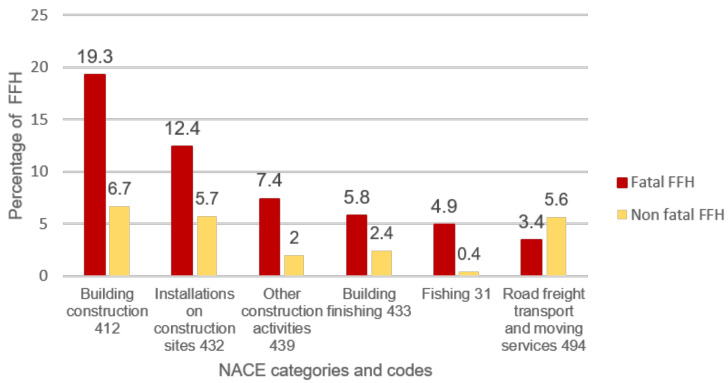
Distribution of Fatal and Non Fatal FFH by NACE.

FFH accidents were distributed among several different categories. However, if only fatal FFH accidents are considered, the total weight of construction groups (NACE CODES = 412,432,439,433) rises from 16.8 percent to 44.9 percent. In Figure 1, the percentage of cited fatal and non-fatal FFH accidents increased. In construction activities, percentage of Fatal FFH accidents increased with respect to their non-fatal accidents. (NACE CODES = 412,432,439,433) rises from 16.8 percent to 44.9 percent. In Figure 1 percentage of Fatal and non Fatal FFH cited increase was illustrated. In construction activities, percentage of Fatal FFH accidents increased with respect to their non-fatal accidents.

### 2.2. Research Design and Variables

The research design was based on the methodology proposed in a previous paper [31] that was applied successfully to other similar studies [34,35,36], and it is described below.

Accident data provided by Spanish labor authorities included 58 variables per each accident. In a preliminary approach, all 58 variables were analyzed through contingency tables. Then, variables with a statistical significance lower than 95 percent were rejected because it was not possible to confirm the existence of more than a random influence for the severity value.

After that, non-rejected variables included in accident reports with the statistical significance were categorized into the following groups:

- Personal variables that show characteristics of the worker affected by the accident, such as sex and age, were included in this category
- Organization variables that describe details about the activities were developed. This group includes worker contract, length of service, company staff and place of the accident.
- Material variables that include injury type and material agent were included
- Material variables include injury type and material agent
- Geographic variables that include the zone of the accident according to their climatic parameters were included.

### 2.3. Statistics Tools

To test the independence of each variable with respect to the severity, contingency tables were created using SPSS 23. Statistical techniques, such as the calculation of chi-square values and corrected standard residuals (CSR), were used. Absolute values of the csr under 1.96 did not reach a statistical significance of 95 percent; thus, it was not possible to confirm the existence of more than a random influence for severity variable. According to the preliminary results, more statistically significant variables were selected for the current research, as shown in Table 2.

Severity values in an accident notification form are based on medical criteria at the following levels: light, serious, very serious or fatal. Accidents without long -term physical damage to the worker were considered light. In the case of serious accidents, more than 120 days of absence at work are expected, but the life of the worker is not at risk, and no disability is experienced by the worker. In contrast, in a very serious accident, for the life of the worker is at risk and/or a disability is linked to the accident. Finally, in fatal accidents, the worker dies as a consequence of accident damage.

Additionally, accident ratios for the severity of accidents were calculated for a deeper analysis. The rates were calculated by dividing the number of accidents chosen in the category studied by the number of total accidents chosen [31,34].

FFH accident rates were calculated by dividing the number of accidents chosen in the group studied by the number of total accidents selected. Then, the total accident rate (TAR) was calculated by dividing the number of total accidents in the group studied by the number of total accidents analyzed. The light accident rate (LAR) was calculated by dividing the number of light accidents in the group studied by the total number of light accidents. The serious accident rate (SAR) was calculated by dividing the number of serious accidents in the group studied by the number of total serious accidents. The very serious accident rate (VSAR) was calculated by dividing the number of very serious accidents in the group studied by the total number of very serious accidents. Finally, the fatal accident rate (FAR) was calculated by dividing the number of fatal accidents in the group studied by the total number of fatal accidents.

It is important to bear in mind that rates obtained are not the common incidence rate (number of accidents per worker exposed) because it was not possible to obtain the number of workers exposed disaggregated in each variable studied. However, all rates obtained are based in the total number of recorded accidents. Then, it is possible to compare calculated rates in a same category. For instance, a higher percentage of males in the total number of accidents can be motivated by a lower number of female workers. However, if the fatal accident rate of men is higher than total accident rate of men, it can be concluded that once the accident happens, men are more likely to suffer fatal consequences.

Statistical results were obtained using the Statistical Package per Social Science (SPSS) version 23.

## 3. Results and Discussion

### 3.1. Personal Factors

#### 3.1.1. Gender of Worker

The results in Table 3 showed that FFH accidents suffered by women are less likely to be fatal than occupational falls suffered by men. The rate of accidents increased with severity in male workers. In the case of females, these rates decreased. In a study about occupational accidents that was not focused only on FFH accidents, it was concluded that male workers had a significantly higher prevalence of fatal occupational injuries than female workers [37]. In the particular case of FFH accidents in construction, previous results concluded that males are more likely to be victims of fatal falls in the construction industry [12]. Gender differences can exist because they faced different tasks and levels of workplace safety risks, especially in traditional occupations for men [38], as construction [39]. Another gender difference is that the feminine dimension showed safer attitudes and a minimized amount of risk-taking, while the masculine dimension showed weak attitudes towards safety and an increased risk propensity [40].

#### 3.1.2. Age Factor

According to the results shown in Table 4, once an accident happens, older workers are more likely to suffer fatal consequences. In the group of workers under 40 years old, their fatal accident rates were lower than their total accident rates; in contrast, workers over 40 showed fatal accident rates higher than their total accident rates, especially the group of workers between 50 and 59 years old (TAR of 22.9% and FAR of 30.3%). These results are aligned with other more general studies about accidents. For instance, in Japan [15] it was detected that occupational accidents are more likely to cause death to workers older than 60, while in countries like Spain, it was concluded that older workers have a higher probability of suffering from severe injuries than younger ones [41]. The same conclusion was reached in Poland [42]. In the particular case of occupational falls from ladders, the conclusion was the same [6]. The majority of existing literature indicates that older workers endured more severe and more costly work-related injuries [43,44], thus, the particular case of FFH accidents does not differ from the trends for accidents in general.

### 3.2. Organization Factors

#### 3.2.1. Worker Contract

Regarding worker contracts (Table 5), self-employed (TAR 4.9%, FAR 7.9%) and sea workers (TAR 0.85,% FAR 6.7) showed the worst rates. Occupational accidents for the self-employed are not a new problem [45]. Research shows that self-employed workers tend to work in dangerous industries with high fatality rates [25,46]. Self-employed workers complete their tasks under precarious conditions, and many studies have reported a positive association between precarious employment and occupational injuries [47].

#### 3.2.2. Length of Service

**Figure 2:**
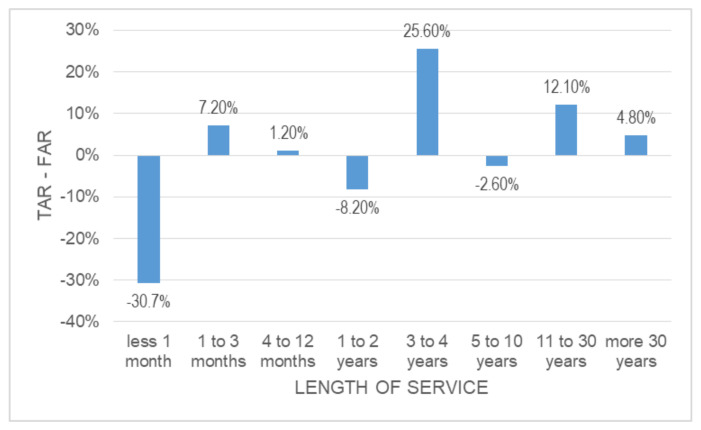
Difference of TAR-FAR based on length of service.

FFH accidents that happen in the first month of service are more likely to be fatal than FFH accidents in other periods, as shown in Figure 2 and Table 6. This factor has been studied in the construction sector by other authors, such as Dong et al. [33]. They found a higher risk of fatal falls for Hispanic workers in their first year of service. Remarkably, the worst rate is concentrated only in the first month of service. Workers with 5–10 years of experience provided the worst rates in similar studies [34]. Then, FFH accidents are significantly influenced by inexperience compared to the rest of the occupational accidents. Specific training programs for inexperienced workers could help to fix this problem [48,49].

#### 3.2.3. Company Size

**Figure 3:**
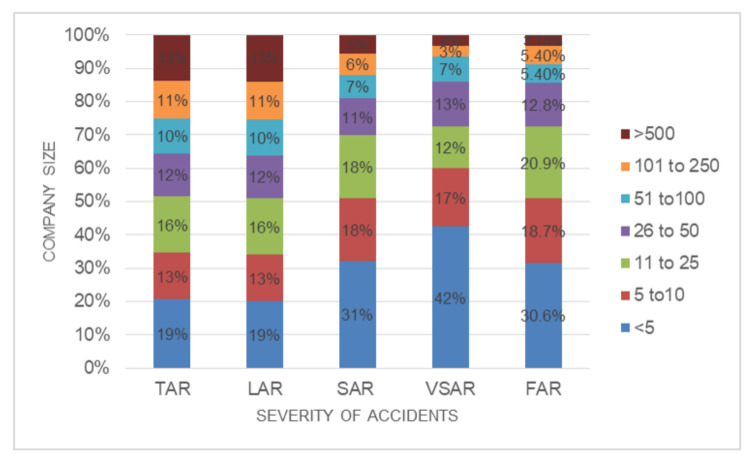
Distribution of severity rates versus company size.

Regarding the company size, the results shown in Figure 3 show that FFH accidents in very small companies are more likely to be fatal (TAR of 19% and FAR of 31%), while this probability decreases in bigger companies. Most authors agree that small companies present worse numbers due to their higher risks [25,50]. Large companies have better safety records than small and very small companies [51,52,53], because smaller companies have fewer resources for safety management [54].

#### 3.2.4. Place of the Accident

Although 74.4% of the FFH accidents happened at the habitual workplace (Table 7), only 51% of fatal falls take place there. In contrast, falls in non-habitual workplaces represented 6.7% of the total falls, but this percentage is close to the third part of fatal falls (FAR of 28.8%). In previous research focused on construction accidents, the number of accidents that occur at a non-habitual site represented less than 11% of the total but was more than double considering fatalities [34,35]. Thus, safety training and safety conditions at non-habitual places are critical for the improvement of fatality rates..

### 3.3. Temporal Factors

The distribution of the accidents throughout the week was analyzed to detect the possibility of the Monday effect [55,56].However, no evidence was found because fall rates were distributed similarly from Monday to Friday. This result is logical because it is very difficult to suffer a fall on Sunday and translate its consequence to Monday; thus, Monday effects are more associated with light musculoskeletal disorders, such as sprains or low back pain. Another temporal variable analyzed was the time of the accident. As a result, significant differences were not found between the distribution and rates of fall accidents in the morning (7–15 h), afternoon (16–23 h), or evening (24–7 h).

### 3.4. Material Factors

#### 3.4.1. Material Agent

Regarding the material agent as the cause of the fall, construction or the surface upper level was involved in nearly half of the total falls and in 65.4% of the fatal falls (Table 8). Another important material agent detected was the transportation system (TAR of 4.3% and FAR of 10.6%).

#### 3.4.2. Part of the Body Injured

Depending on the part of the body affected (Table 9), severity rates of the falls changed. Multiple part injuries were only 10.3% of the accidents, but they were the biggest percentage of deaths (FAR of 61.4%). In contrast, lower and upper extremities were injured 67.1% of the time.

### 3.5. Geographic Factors

The geographical location of an accident can be influenced by different factors, such as population, culture, economy or weather conditions (Table 10). Considering only climatic conditions, we observed that in provinces with an average temperature lower than 13.1 degrees Celsius, FFH accidents were more likely to be fatal. In contrast, a higher temperature showed better results. These results seem to indicate that continental weather with more wetness, ice and snow influence the consequences of falls. Other authors found a strong relationship between heat and incidence rates for work injuries [57], but they did not find evidence of higher severity at higher temperatures. Additional climatic variables should be studied in more detail to obtain more results that are significant.

## 4. Conclusions

This research showed that the severity of FFH occupational accidents was related to the variables studied. The most remarkable result was the difference found based on the gender of the workers. Results showed that after a FFH accident happens, a male worker is more likely to suffer a fatal accident than a female worker. These higher rates for men can be motivated by their activities at work and their attitudes regarding safety and occupational risks. Therefore, preventive measures, such as safety training, should be adapted to the gender of the workers and their tasks at the workplace. The age of the worker was indicated to be another relevant variable. Older workers were more likely to suffer fatal consequences once an accident happened. Therefore, senior workers should be specially considered when preventive and safety plans have been designed. Regarding worker contract, self-employed workers showed the worst fatality rates. Small and very small companies presented negative results, too. Therefore, these groups of workers should be protected by providing them with additional safety tools by contractors and labor authorities because frequently these companies do not have the economic resources for safety management. The experience of the worker was highlighted as another relevant variable, as the worst rates were concentrated in the first month of service. Thus, extra effort should be concentrated in safety training programs for inexperienced workers. The non-habitual workplace was identified as another relevant factor of fatal FFH accidents. Finally, geographical factors showed that regions with higher temperatures obtained statistically more significant results. A better knowledge of FFH accidents will improve safety conditions. The identification of the main variables in FFH accidents is the first step to reducing occupational accidents and their consequences. Specific safety training can be developed that considers specific requirements for each category of workers and for each type of company and organization. The workers should be trained and informed about their specific risk depending on the variables analyzed.

### 4.1. Limitation of the Study

Although in Spain it is compulsory to notify occupational accidents to the labor authorities, it is possible that some accidents that happened were not reported. Thus, a possible bias caused by underreported accidents might be considered. In addition, only variables included in Official Accident Notification Form were analyzed. Reports of fatal accidents include additional information, but these reports were not possible to obtain.

### 4.2. Future Research

Results from the current study can help to identify suitable mitigating actions in future safety planning. Thus, the development of safety programs based on the current results and an analysis of their effectiveness in the workplace should be explored. A study of the multiple effects of several variables simultaneously on the severity of FFH accidents might provide complementary results to those obtained in the current research.

A combination of the analysis of official data with surveillance and interviews in the workplace in future research could improve the safety management and the risk of FFH accidents.

## Figures and Tables

**Table 1:** Distribution of FFH accidents by NACE code.

NACE Code	Description	N° of FFH	%
412	Building construction	19,351	6.7
432	Installations on construction sites	16,594	5.7
494	Road freight transport and moving services	16,356	5.6
841	Public Administration and social policy	15,559	5.4
812	Cleaning activities	13,654	4.7
561	Restaurants and food stalls	7663	2.6
861	Hospital activities	7610	2.6
433	Building finishing	6945	2.4
12	Perennial crops	6903	2.4
471	Retail trade in non-specialized stores	6573	2.3
477	Retail sale of other items in specialized stores	5955	2
563	Beverage establishments	5906	2
439	Other specialized construction activities	5818	2
11	Non-perennial crops	5455	1.9
463	Wholesale of food, beverages and tobacco	4935	1.7
	Total	145,277	50

**Table 2:** Selection of variables analyzed according to their statistical significance.

Variable	Chi Square	d.f	Sig
Gender	1536	3	0.000
Age	837	27	0.000
Contract	590	15	0.000
Length of service	195	21	0.000
Staff	1864	21	0.000
Place	3159	9	0.000
Material agent	391	18	0.000
Part of the body	837	27	0.000
Geographic	1801	153	0.000

**Table 3:** Gender of workers.

Gender	#	TAR	#	LAR	#	SAR	#	VSAR	#	FAR
Male	206,614	71.1%	198,504	70.5%	7271	88.6%	301	95.3%	538	96.9%
Female	83,969	28.9%	82,999	29.5%	938	11.4%	15	4.7%	17	3.1%
Total	290,583	100.0%	281,503	100.0%	8209	100.0%	316	100.0%	555	100.0%

**Table 4:** Age of injuried.

Age	#	TAR	#	LAR	#	SAR	#	VSAR	#	FAR
under 16	2 *	0.0%	2 *	0.0%	0 *	0.0%	0 *	0.0%	0 *	0.0%
16–19	2669	0.9%	2624	0.9%	43	0.5%	1 *	0.3%	1 *	0.2%
20–24	15,825	5.4%	15,625	5.6%	184	2.2%	4	1.3%	12	2.2%
25–29	27,530	9.5%	27,061	9.6%	418	5.1%	24 *	7.6%	27	4.9%
30–39	78,376	27.00%	76,404	27.10%	1787	21.80%	73 *	23.10%	112	20.20%
40–49	84,021	28.90%	81,039	28.80%	2694	32.80%	106 *	33.50%	182	32.80%
50–59	66,484	22.90%	63,770	22.70%	2457	29.90%	89	28.20%	168	30.30%
60–65	15,060	5.20%	14,392	5.10%	599	7.30%	19	6.00%	50	9.00%
65–70	549	0.20%	24	0.20%	24	0.30%	0 *	0.00%	2 *	0.40%
over 70	67 *	0.00%	3 *	0.00%	3 *	0.00%	0 *	0.00%	1	0.20%
Total	290,514	100%	280,939	100%	8206	100%	112	100%	552	100%

* Absolute value of the csr under 1.96.

**Table 5:** Workers distribution by contract.

Worker Contract	#	TAR	#	LAR	#	SAR	#	VSAR	#	FAR
Employed	269,012	92.58%	261,015	92.72%	7263	88.48%	265	83.86%	469	84.50%
Self employed	14171	4.88%	13,407	4.76%	682	8.31%	38	12.03%	44	7.93%
Agriculture employed	4125	1.42%	4002 *	1.42%	116 *	1.41%	3 *	0.95%	4 *	0.72%
Agriculture self employed	553	0.19%	502	0.18%	47	0.57%	3	0.95%	1 *	0.18%
Sea worker	2464	0.85%	2324	0.83%	97	1.18%	6	1.90%	37	6.67%
Mining and coal	258	0.09%	253 *	0.09%	4 *	0.05%	1 *	0.32%	0 *	0.00%
Total	290,583	1	281,503	1	8209	1	316	1	555	1

* Absolute value of the csr under 1.96.

**Table 6:** Length of service.

Lenght of Service	#	TAR	#	LAR	#	SAR	#	VSAR	#	FAR
less 1 month	44,543	15.3%	42,739	15.2%	1632	19.9%	61	19.3%	111	20.0%
1–3 months	24,070	8.3%	23,352	8.3%	646 *	7.9%	29 *	9.2%	43 *	7.7%
4–12 months	46,957	16.2%	45,553	16.2%	1264	15.4%	51 *	16.1%	89 *	16.0%
1–2 years	28,076	9.7%	27,268	9.7%	719	8.8%	31 *	9.8%	58 *	10.5%
3–4 years	35,167	12.10%	34,192	12.10%	890	10.80%	35 *	11.10%	50 *	9.00%
5–10 years	55,085	19.00%	53,556	19.00%	1375	16.70%	46	14.60%	108 *	19.50%
11–30 years	50,444 *	17.40%	48,834 *	17.30%	1465 *	17.80%	60 *	19.00%	85 *	15.30%
more 30 years	6241	2.10%	6009	2.10%	218	2.70%	3 *	0.90%	11 *	2.00%
Total	290,583	100.00%	8209	100.00%	8209	100.00%	316	100.00%	555	100.00%

* Absolute value of the csr under 1.96.

**Table 7:** Place of the accident.

Place	#	TAR	#	LAR	#	SAR	#	VSAR	#	FAR
Habitual worksite	216,261	74.4%	210,521	74.8%	5291	64.5%	166	52.5%	283	51.0%
On the way	24,521	8.4%	23,387	8.3%	974	11.9%	59	18.7%	101	18.2%
Going or backing home	30,202	10.4%	29,787	10.6%	400	4.9%	4	1.3%	11	2.0%
Non-habitual worksite	19,599	6.7%	17,808	6.3%	1544	18.8%	87	27.5%	160	28.8%

**Table 8:** Material Agent.

Material Agent	#	TAR	#	LAR	#	SAR	#	VSAR	#	FAR
Construction or surface-same level	35,649	12.3%	34,805	12.4%	803	9.8%	17	5.4%	24	4.3%
Construction or surface-upper level	133,718	46.0%	129,297	45.9%	3869	47.1%	189	59.8%	363	65.4%
Machine or device manual	5643	1.9%	5405	1.9%	224	2.7%	9 *	2.8%	5 *	0.9%
Machine or device static	2680	0.90%	2606	0.90%	59	0.70%	2 *	0.60%	13	2.30%
Transportation systems	12,573	4.30%	12,029	4.30%	461	5.60%	24	7.60%	59	10.60%
Office staff	4873	1.70%	4805	1.70%	65	0.80%	0	0.00%	3	0.50%
Other causes	95,447	32.8%	92,556	32.9%	2728	33.3%	75	24%	88	16%
Total	290,583	100.00%	281,503	100.00%	8209	100.00%	316	100.00%	555	100.00%

* Absolute value of the csr under 1.96.

**Table 9:** Part of body injured.

Part of the Body Injured	#	TAR	#	LAR	#	SAR	#	VSAR	#	FAR
No information	1499	0.5%	1452	0.5%	47 *	0.6%	0 *	0.0%	0 *	0.0%
Head	9121	3.1%	8023	2.9%	799	9.7%	123	38.9%	176	31.7%
Neck	4243	1.5%	4149	1.5%	83	1.0%	6 *	1.9%	5 *	0.9%
Back	29,764	10.2%	28,775	10.2%	965	11.8%	22	7.0%	2	0.4%
Trunk	21,047	7.20%	20,440	7.30%	563	6.90%	16 *	5.10%	28	5.00%
Upper extremity	71,726	24.70%	70,272	25.00%	1448	17.60%	6	1.90%	0	0.00%
Lower exremity	123,269	42.40%	120,301	42.70%	1465 *	35.80%	27	8.50%	3	0.50%
Multiple parts	29,914	10.30%	28,091	10.00%	1366	16.60%	116	36.70%	341	61.40%
Total	290,583	100.00%	8209	100.00%	8209	100.00%	316	100.00%	555	100.00%

* Absolute value of the csr under 1.96.

**Table 10:** Difference between TAR-LAR according to geographic location.

County	TAR-FAR	Temp (ºC)	Rain (mm)
Sevilla	0.0%	17.3	584
Madrid	38.4%	13.1	537
Barcelona	32.0%	13.1	690
Coruña	−80.8%	12.7	1646
Burgos	−125.0%	10.5	655
Pontevedra	−125.0%	13	1604
Segovia	−200.0%	11.2	538
Teruel	−333.3%	11.2	479

## Abbreviations

The following abbreviations are used in this manuscript:

FFH	Falls from heights
csr	corrected standard residuals
